# {μ-2-[4-(1,3-Benzothia­zol-2-yl)phen­yl]-2-aza­propane-1,3-dithiol­ato-κ^4^ *S*,*S*′:*S*,*S*′}bis­[tricarbonyl­iron(I)]

**DOI:** 10.1107/S1600536812003753

**Published:** 2012-02-04

**Authors:** Shang Gao, Qian Duan, Da-yong Jiang

**Affiliations:** a School of Materials Science and Engineering, Changchun University of Science and Technology, No. 7989 Weixing Road, Changchun 130022, People’s Republic of China

## Abstract

The title compound, [Fe_2_(C_15_H_12_N_2_S_3_)(CO)_6_], was prepared as an aza­dithiol­atodiiron model for the active site of [FeFe]-hydrogenase. The Fe_2_S_2_ core adopts a butterfly shape, with each metal having a pseudo square-pyramidal geometry. The *N*-substituted aza­dithiol­ate is μ_2_-κ^4^ *S*,*S*′:*S*,*S*′-coordinated to the Fe(CO)_3_ moieties to form two fused six-membered rings with different conformations. The sum of the C—N—C angles around the N atom [356.85 (15)°] indicates a flattening of the trigonal–pyramidal geometry about the N atom and an increase in the degree of *sp* ^2^-hybridization.

## Related literature

For reviews of hydrogenases, see: Cammack (1999 ▶); Evans & Pickett (2003 ▶). For the synthesis and structures of models for the active site of Fe-only hydrogenases, see: Lawrence *et al.* (2001 ▶); Song *et al.* (2005 ▶); Liu & Xiao (2011 ▶); Yin *et al.* (2011 ▶); For structures of Fe-only hydrogenases, see: Nicolet *et al.* (1999 ▶); Peters *et al.* (1998 ▶).
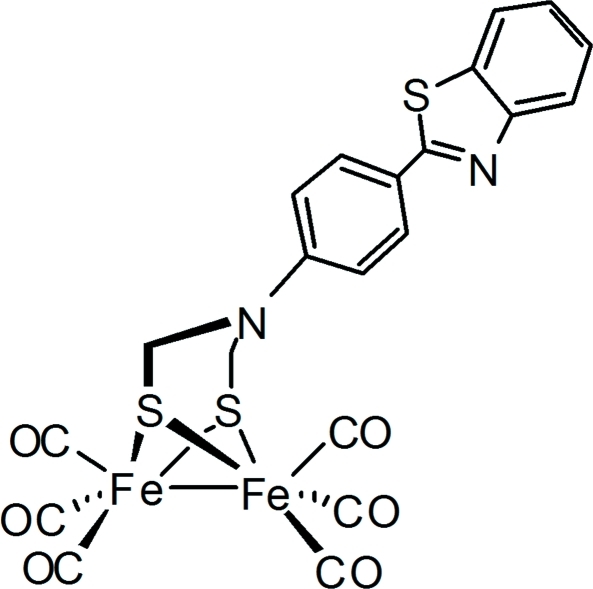


## Experimental

### 

#### Crystal data

[Fe_2_(C_15_H_12_N_2_S_3_)(CO)_6_]
*M* *_r_* = 596.21
Monoclinic, 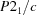

*a* = 13.8606 (12) Å
*b* = 7.6900 (7) Å
*c* = 21.5052 (19) Å
β = 92.447 (1)°
*V* = 2290.1 (4) Å^3^
*Z* = 4
Mo *K*α radiation
μ = 1.58 mm^−1^
*T* = 273 K
0.45 × 0.22 × 0.10 mm

#### Data collection

Bruker APEXII CCD area-detector diffractometer
Absorption correction: multi-scan (*SADABS*; Bruker, 2001 ▶) *T*_min_ = 0.536, *T*_max_ = 0.858
12350 measured reflections
4509 independent reflections
4124 reflections with *I* > 2σ(*I*)
*R*_int_ = 0.020

#### Refinement

*R*[*F* ^2^ > 2σ(*F* ^2^)] = 0.023
*wR*(*F* ^2^) = 0.063
*S* = 1.04
4509 reflections
307 parameters
H-atom parameters constrained
Δρ_max_ = 0.28 e Å^−3^
Δρ_min_ = −0.23 e Å^−3^

### 

Data collection: *APEX2* (Bruker, 2005 ▶); cell refinement: *SAINT-Plus* (Bruker, 2001 ▶); data reduction: *SAINT-Plus*; program(s) used to solve structure: *SHELXS97* (Sheldrick, 2008 ▶); program(s) used to refine structure: *SHELXL97* (Sheldrick, 2008 ▶); molecular graphics: *SHELXTL* (Sheldrick, 2008 ▶); software used to prepare material for publication: *SHELXTL*.

## Supplementary Material

Crystal structure: contains datablock(s) global, I. DOI: 10.1107/S1600536812003753/mw2048sup1.cif

Structure factors: contains datablock(s) I. DOI: 10.1107/S1600536812003753/mw2048Isup2.hkl

Additional supplementary materials:  crystallographic information; 3D view; checkCIF report

## Figures and Tables

**Fig. 1.**
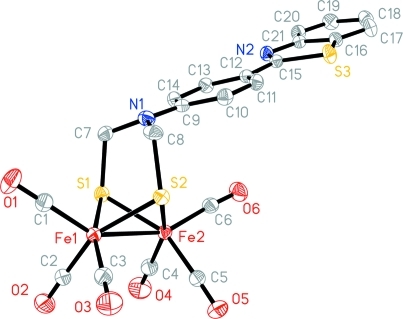
The molecular structure of the title compound with displacement ellipsoids drawn at the 30% probability level and H-atoms omitted.

## supplementary crystallographic information

### Comment

Recently [FeFe]-hydrogenases ([FeFe]Hases) have received more attention than other types of hydrogenases due to their much higher efficiency in hydrogen production (Cammack, 1999; Evans & Pickett, 2003). Crystallographic and IR spectroscopy studies revealed the active site of [FeFe]Hases (so-called H-cluster) to contain a butterfly Fe_2_S_2_ subunit (Peters *et al.*, 1998) and a variety of transition metal sulfides have been prepared as models of the diiron subunit of the H-cluster (Song *et al.*, 2005; Yin *et al.*, 2011; Liu & Xiao, 2011). We have synthesized the title compound as a structural model for the active site of [FeFe]Hases and report its crystal structure.

In the title compound, the Fe_2_S_2_ core adopts a butterfly framework with each metal having a pseudo square-pyramidal geometry. The length of the Fe—Fe bond [2.5036 (4) Å] is slightly shorter than those in the structures of natural enzymes (*ca* 2.6 Å) (Peters *et al.*, 1998; Nicolet *et al.*, 1999). The structure features two fused six-membered rings: Fe1—S1—C7—N1—C8—S2 which adopts a chair conformation and Fe2—S1—C7—N1—C8—S2 which adopts a boat conformation. The sum of the C—N—C angles around N1 atom is 356.85 (15)° implying a noticeable flattening of the trigonal pyramidal geometry about N1 and an increase in the degree of *p*-π conjugation between the substituted phenyl ring and the *p*-orbital of the bridging N atom. The distance of N1 from the plane defined by C7, C8 and C9 is 0.146 (2) Å and the dihedral angle between this plane and the mean plane of the adjacent phenyl ring is 2.5 (1)°. The substituted phenyl ring attached to N1 lies in an axial position relative to the metalloheterocycle and slants towards the Fe_2_(CO)_3_ unit. As a result, the C1—Fe1—Fe2 angle is enlarged by *ca* 7° compared with the C6—Fe2—Fe1 angle.

The molecular structure of the title compound is shown in Fig.1.

### Experimental

All reactions and operations related to the title compound were carried out under a dry, prepurified nitrogen atmosphere with standard Schlenk techniques. All solvents were dried and distilled prior to use according to standard methods. The starting material *N*,*N*'-bis(chloromethyl)-(4-benzothiazole)-phenylamine was prepared in 50% yield from 4-benzothiazole-phenylamine (Lawrence *et al.*, 2001). A degassed solution of (µ-S_2_)Fe_2_(CO)_6_ (1.38 g, 4.0 mmol) in 30 ml dry THF was cooled to 195 K. LiEt_3_BH (1 *M* solution in THF, 8.0 ml, 8.0 mmol) was dropped into the above solution by syringe over 30 min. *N*,*N*'-bis(chloromethyl)-(4-benzothiazole)-phenylamine (2.6 g, 8.0 mmol) was added to the resulting dark green solution causing an immediate change in color to dark red. The mixture was stirred for 2 h at 195 K and another 1 h at room temperature. The solvent was removed on a rotary evaporator and the crude product was purified by column chromatography (silica, 20% dichloromethane in hexane as eluent) to give a red solid (1.37 g, 57%). Recrystallization from a CH_2_Cl_2_/hexane solution afforded crystals of the title compound suitable for X-ray study.

### Refinement

The H atoms attached to C were placed in geometrically calculated positions (C—H = 0.93–0.97 Å) and refined as riding, with *U*_iso_(H) = 1.2*U*_eq_(C).

### Crystal data

**Table:**

[Fe_2_(C_15_H_12_N_2_S_3_)(CO)_6_]	*F*(000) = 1200
*M**_r_* = 596.21	*D*_x_ = 1.729 Mg m^−^^3^
Monoclinic, *P*2_1_/*c*	Mo *K*α radiation, λ = 0.71073 Å
Hall symbol: -P 2ybc	Cell parameters from 8061 reflections
*a* = 13.8606 (12) Å	θ = 2.4–28.9°
*b* = 7.6900 (7) Å	µ = 1.58 mm^−^^1^
*c* = 21.5052 (19) Å	*T* = 273 K
β = 92.447 (1)°	Parallelepiped, red
*V* = 2290.1 (4) Å^3^	0.45 × 0.22 × 0.10 mm
*Z* = 4	

### Data collection

**Table:**

Bruker APEXII CCD area-detector diffractometer	4509 independent reflections
Radiation source: fine-focus sealed tube	4124 reflections with *I* > 2σ(*I*)
Graphite monochromator	*R*_int_ = 0.020
φ and ω scans	θ_max_ = 26.0°, θ_min_ = 2.4°
Absorption correction: multi-scan (*SADABS*; Bruker, 2001)	*h* = −17→17
*T*_min_ = 0.536, *T*_max_ = 0.858	*k* = −9→7
12350 measured reflections	*l* = −23→26

### Refinement

**Table:**

Refinement on *F*^2^	0 restraints
Least-squares matrix: full	H-atom parameters constrained
*R*[*F*^2^ > 2σ(*F*^2^)] = 0.023	*w* = 1/[σ^2^(*F*_o_^2^) + (0.0343*P*)^2^ + 0.6533*P*] where *P* = (*F*_o_^2^ + 2*F*_c_^2^)/3
*wR*(*F*^2^) = 0.063	(Δ/σ)_max_ = 0.001
*S* = 1.04	Δρ_max_ = 0.28 e Å^−^^3^
4509 reflections	Δρ_min_ = −0.23 e Å^−^^3^
307 parameters	

### Special details

**Table:**

Geometry. All e.s.d.'s (except the e.s.d. in the dihedral angle between two l.s. planes) are estimated using the full covariance matrix. The cell e.s.d.'s are taken into account individually in the estimation of e.s.d.'s in distances, angles and torsion angles; correlations between e.s.d.'s in cell parameters are only used when they are defined by crystal symmetry. An approximate (isotropic) treatment of cell e.s.d.'s is used for estimating e.s.d.'s involving l.s. planes.
Refinement. Refinement of *F*^2^ against ALL reflections. The weighted *R*-factor *wR* and goodness of fit *S* are based on *F*^2^, conventional *R*-factors *R* are based on *F*, with *F* set to zero for negative *F*^2^. The threshold expression of *F*^2^ > σ(*F*^2^) is used only for calculating *R*-factors(gt) *etc*. and is not relevant to the choice of reflections for refinement. *R*-factors based on *F*^2^ are statistically about twice as large as those based on *F*, and *R*-factors based on ALL data will be even larger.

### Fractional atomic coordinates and isotropic or equivalent isotropic displacement parameters (Å^2^)

**Table:**

	*x*	*y*	*z*	*U*_iso_*/*U*_eq_	
Fe2	0.110403 (16)	0.28234 (3)	0.149643 (10)	0.03265 (8)	
Fe1	0.139110 (16)	0.58723 (3)	0.114026 (11)	0.03417 (8)	
S1	0.25452 (3)	0.37829 (6)	0.116610 (19)	0.03648 (10)	
S2	0.11928 (3)	0.51324 (5)	0.214607 (19)	0.03527 (10)	
S3	0.30972 (4)	−0.29556 (7)	0.43894 (2)	0.04817 (13)	
C15	0.37264 (11)	−0.2378 (2)	0.37251 (8)	0.0354 (4)	
N1	0.30863 (11)	0.3996 (2)	0.24269 (7)	0.0407 (3)	
C9	0.31954 (11)	0.2394 (2)	0.27348 (8)	0.0347 (4)	
N2	0.43589 (10)	−0.3512 (2)	0.35595 (7)	0.0392 (3)	
C16	0.37529 (13)	−0.4878 (2)	0.44399 (8)	0.0410 (4)	
C7	0.34275 (12)	0.4258 (3)	0.18192 (9)	0.0432 (4)	
H7A	0.3992	0.3532	0.1772	0.052*	
H7B	0.3633	0.5459	0.1785	0.052*	
C1	0.21836 (13)	0.7719 (2)	0.11426 (9)	0.0434 (4)	
C12	0.35215 (11)	−0.0756 (2)	0.33922 (7)	0.0345 (3)	
C20	0.49798 (14)	−0.6393 (3)	0.38954 (9)	0.0463 (4)	
H20A	0.5398	−0.6466	0.3570	0.056*	
C11	0.28990 (12)	0.0515 (2)	0.36067 (8)	0.0402 (4)	
H11A	0.2586	0.0321	0.3974	0.048*	
C8	0.23726 (14)	0.5242 (2)	0.25908 (9)	0.0432 (4)	
H8A	0.2640	0.6396	0.2538	0.052*	
H8B	0.2254	0.5101	0.3029	0.052*	
C6	0.13012 (13)	0.0967 (2)	0.20065 (8)	0.0415 (4)	
C17	0.37132 (16)	−0.6217 (3)	0.48743 (9)	0.0529 (5)	
H17A	0.3293	−0.6159	0.5200	0.063*	
C3	0.02675 (14)	0.7039 (3)	0.11762 (9)	0.0476 (4)	
C19	0.49426 (15)	−0.7706 (3)	0.43218 (10)	0.0524 (5)	
H19A	0.5343	−0.8668	0.4288	0.063*	
C10	0.27360 (12)	0.2049 (2)	0.32888 (9)	0.0412 (4)	
H10A	0.2315	0.2867	0.3444	0.049*	
C18	0.43067 (16)	−0.7617 (3)	0.48100 (10)	0.0555 (5)	
H18A	0.4288	−0.8526	0.5095	0.067*	
C14	0.38064 (13)	0.1106 (2)	0.25110 (8)	0.0401 (4)	
H14A	0.4110	0.1284	0.2139	0.048*	
C2	0.12325 (13)	0.5549 (3)	0.03178 (9)	0.0475 (4)	
O6	0.13791 (12)	−0.0230 (2)	0.23068 (7)	0.0631 (4)	
C21	0.43879 (12)	−0.4948 (2)	0.39519 (8)	0.0370 (4)	
O4	0.09231 (14)	0.0933 (2)	0.03168 (8)	0.0794 (5)	
C13	0.39634 (12)	−0.0421 (2)	0.28349 (8)	0.0383 (4)	
H13A	0.4376	−0.1251	0.2677	0.046*	
C4	0.09890 (15)	0.1660 (3)	0.07745 (9)	0.0479 (4)	
O2	0.11222 (12)	0.5315 (3)	−0.02042 (7)	0.0755 (5)	
O3	−0.04242 (12)	0.7811 (2)	0.12089 (9)	0.0775 (5)	
O5	−0.10051 (10)	0.2857 (2)	0.15324 (7)	0.0628 (4)	
C5	−0.01899 (13)	0.2869 (2)	0.15329 (8)	0.0417 (4)	
O1	0.27005 (11)	0.8864 (2)	0.11324 (9)	0.0698 (5)	

### Atomic displacement parameters (Å^2^)

**Table:**

	*U*^11^	*U*^22^	*U*^33^	*U*^12^	*U*^13^	*U*^23^
Fe2	0.03394 (13)	0.03136 (14)	0.03265 (13)	−0.00374 (9)	0.00159 (9)	−0.00151 (9)
Fe1	0.03240 (13)	0.03442 (14)	0.03560 (14)	−0.00048 (9)	0.00050 (9)	0.00395 (10)
S1	0.0352 (2)	0.0389 (2)	0.0358 (2)	0.00191 (17)	0.00669 (16)	−0.00009 (17)
S2	0.0368 (2)	0.0348 (2)	0.0344 (2)	0.00106 (17)	0.00365 (16)	−0.00375 (16)
S3	0.0525 (3)	0.0520 (3)	0.0413 (2)	0.0123 (2)	0.0164 (2)	0.0083 (2)
C15	0.0308 (8)	0.0442 (10)	0.0313 (8)	−0.0017 (7)	0.0010 (6)	−0.0003 (7)
N1	0.0400 (8)	0.0405 (8)	0.0412 (8)	0.0023 (6)	−0.0049 (6)	0.0018 (6)
C9	0.0299 (8)	0.0394 (9)	0.0343 (8)	−0.0017 (7)	−0.0050 (6)	−0.0006 (7)
N2	0.0357 (7)	0.0432 (8)	0.0392 (8)	0.0021 (6)	0.0062 (6)	0.0036 (6)
C16	0.0427 (9)	0.0442 (10)	0.0362 (9)	0.0011 (8)	0.0029 (7)	0.0023 (8)
C7	0.0328 (9)	0.0457 (10)	0.0507 (11)	−0.0039 (7)	−0.0031 (7)	0.0118 (8)
C1	0.0371 (9)	0.0390 (10)	0.0544 (11)	0.0054 (8)	0.0040 (8)	0.0045 (8)
C12	0.0309 (8)	0.0393 (9)	0.0332 (8)	−0.0012 (7)	−0.0008 (6)	−0.0012 (7)
C20	0.0431 (10)	0.0481 (11)	0.0482 (11)	0.0048 (8)	0.0056 (8)	0.0013 (9)
C11	0.0360 (9)	0.0483 (10)	0.0367 (9)	0.0004 (8)	0.0079 (7)	0.0013 (8)
C8	0.0514 (10)	0.0359 (9)	0.0413 (9)	0.0009 (8)	−0.0120 (8)	−0.0048 (8)
C6	0.0448 (10)	0.0385 (10)	0.0408 (9)	−0.0051 (8)	−0.0030 (7)	−0.0046 (8)
C17	0.0657 (13)	0.0521 (12)	0.0416 (10)	0.0038 (10)	0.0120 (9)	0.0083 (9)
C3	0.0434 (10)	0.0473 (11)	0.0520 (11)	0.0016 (9)	0.0009 (8)	0.0083 (9)
C19	0.0548 (12)	0.0462 (11)	0.0559 (12)	0.0107 (9)	0.0002 (9)	0.0026 (9)
C10	0.0344 (9)	0.0442 (10)	0.0454 (10)	0.0055 (7)	0.0057 (7)	−0.0019 (8)
C18	0.0711 (14)	0.0467 (12)	0.0484 (12)	0.0032 (10)	0.0003 (10)	0.0122 (9)
C14	0.0429 (9)	0.0455 (10)	0.0323 (8)	0.0001 (8)	0.0061 (7)	−0.0010 (7)
C2	0.0416 (10)	0.0589 (12)	0.0418 (11)	−0.0057 (9)	−0.0003 (8)	0.0099 (9)
O6	0.0816 (11)	0.0460 (8)	0.0606 (9)	−0.0110 (8)	−0.0084 (8)	0.0136 (7)
C21	0.0344 (8)	0.0397 (9)	0.0367 (9)	−0.0017 (7)	−0.0002 (7)	0.0016 (7)
O4	0.1012 (14)	0.0845 (13)	0.0520 (9)	−0.0165 (10)	−0.0026 (9)	−0.0272 (9)
C13	0.0372 (9)	0.0414 (9)	0.0366 (9)	0.0056 (7)	0.0049 (7)	−0.0019 (7)
C4	0.0543 (11)	0.0443 (11)	0.0449 (11)	−0.0076 (9)	−0.0002 (8)	−0.0045 (9)
O2	0.0719 (11)	0.1161 (15)	0.0383 (9)	−0.0164 (10)	−0.0014 (7)	0.0050 (9)
O3	0.0480 (9)	0.0859 (13)	0.0990 (14)	0.0244 (9)	0.0068 (8)	0.0107 (10)
O5	0.0376 (8)	0.0841 (11)	0.0664 (10)	−0.0050 (7)	0.0000 (6)	0.0041 (8)
C5	0.0431 (10)	0.0420 (10)	0.0398 (9)	−0.0055 (8)	−0.0008 (7)	0.0014 (8)
O1	0.0516 (9)	0.0480 (9)	0.1105 (14)	−0.0123 (7)	0.0129 (9)	0.0029 (9)

### Geometric parameters (Å, º)

**Table:**

Fe2—C4	1.793 (2)	C7—H7B	0.9700
Fe2—C5	1.7989 (19)	C1—O1	1.136 (2)
Fe2—C6	1.8140 (19)	C12—C13	1.393 (2)
Fe2—S2	2.2594 (5)	C12—C11	1.395 (2)
Fe2—S1	2.2717 (5)	C20—C19	1.366 (3)
Fe2—Fe1	2.5036 (4)	C20—C21	1.389 (3)
Fe1—C2	1.790 (2)	C20—H20A	0.9300
Fe1—C1	1.7954 (19)	C11—C10	1.377 (3)
Fe1—C3	1.802 (2)	C11—H11A	0.9300
Fe1—S2	2.2647 (5)	C8—H8A	0.9700
Fe1—S1	2.2663 (5)	C8—H8B	0.9700
S1—C7	1.8589 (18)	C6—O6	1.127 (2)
S2—C8	1.8607 (18)	C17—C18	1.366 (3)
S3—C16	1.7360 (19)	C17—H17A	0.9300
S3—C15	1.7622 (17)	C3—O3	1.133 (2)
C15—N2	1.298 (2)	C19—C18	1.401 (3)
C15—C12	1.460 (2)	C19—H19A	0.9300
N1—C9	1.404 (2)	C10—H10A	0.9300
N1—C7	1.423 (2)	C18—H18A	0.9300
N1—C8	1.432 (2)	C14—C13	1.378 (2)
C9—C10	1.400 (2)	C14—H14A	0.9300
C9—C14	1.402 (2)	C2—O2	1.141 (2)
N2—C21	1.389 (2)	O4—C4	1.133 (2)
C16—C17	1.393 (3)	C13—H13A	0.9300
C16—C21	1.399 (2)	O5—C5	1.130 (2)
C7—H7A	0.9700		
			
C4—Fe2—C5	89.75 (9)	C21—C16—S3	109.31 (13)
C4—Fe2—C6	97.84 (9)	N1—C7—S1	115.61 (12)
C5—Fe2—C6	96.54 (8)	N1—C7—H7A	108.4
C4—Fe2—S2	158.12 (7)	S1—C7—H7A	108.4
C5—Fe2—S2	89.18 (6)	N1—C7—H7B	108.4
C6—Fe2—S2	103.99 (6)	S1—C7—H7B	108.4
C4—Fe2—S1	86.46 (6)	H7A—C7—H7B	107.4
C5—Fe2—S1	154.01 (6)	O1—C1—Fe1	178.08 (19)
C6—Fe2—S1	109.45 (6)	C13—C12—C11	117.11 (16)
S2—Fe2—S1	84.991 (17)	C13—C12—C15	119.76 (15)
C4—Fe2—Fe1	102.22 (7)	C11—C12—C15	123.13 (15)
C5—Fe2—Fe1	99.59 (6)	C19—C20—C21	119.52 (18)
C6—Fe2—Fe1	154.19 (6)	C19—C20—H20A	120.2
S2—Fe2—Fe1	56.499 (14)	C21—C20—H20A	120.2
S1—Fe2—Fe1	56.412 (13)	C10—C11—C12	121.78 (16)
C2—Fe1—C1	99.31 (9)	C10—C11—H11A	119.1
C2—Fe1—C3	92.38 (9)	C12—C11—H11A	119.1
C1—Fe1—C3	97.81 (9)	N1—C8—S2	116.38 (12)
C2—Fe1—S2	153.38 (7)	N1—C8—H8A	108.2
C1—Fe1—S2	107.17 (7)	S2—C8—H8A	108.2
C3—Fe1—S2	86.77 (6)	N1—C8—H8B	108.2
C2—Fe1—S1	89.01 (7)	S2—C8—H8B	108.2
C1—Fe1—S1	97.45 (6)	H8A—C8—H8B	107.3
C3—Fe1—S1	164.24 (7)	O6—C6—Fe2	176.05 (17)
S2—Fe1—S1	84.996 (17)	C18—C17—C16	118.27 (19)
C2—Fe1—Fe2	99.09 (7)	C18—C17—H17A	120.9
C1—Fe1—Fe2	147.69 (6)	C16—C17—H17A	120.9
C3—Fe1—Fe2	107.70 (6)	O3—C3—Fe1	177.9 (2)
S2—Fe1—Fe2	56.300 (13)	C20—C19—C18	120.66 (19)
S1—Fe1—Fe2	56.619 (14)	C20—C19—H19A	119.7
C7—S1—Fe1	108.66 (6)	C18—C19—H19A	119.7
C7—S1—Fe2	112.74 (6)	C11—C10—C9	120.92 (16)
Fe1—S1—Fe2	66.969 (15)	C11—C10—H10A	119.5
C8—S2—Fe2	112.15 (6)	C9—C10—H10A	119.5
C8—S2—Fe1	109.86 (7)	C17—C18—C19	121.02 (19)
Fe2—S2—Fe1	67.201 (15)	C17—C18—H18A	119.5
C16—S3—C15	89.40 (8)	C19—C18—H18A	119.5
N2—C15—C12	124.02 (15)	C13—C14—C9	120.90 (16)
N2—C15—S3	114.61 (13)	C13—C14—H14A	119.6
C12—C15—S3	121.36 (12)	C9—C14—H14A	119.6
C9—N1—C7	121.62 (15)	O2—C2—Fe1	178.7 (2)
C9—N1—C8	122.20 (15)	N2—C21—C20	125.75 (16)
C7—N1—C8	113.03 (15)	N2—C21—C16	114.95 (16)
C10—C9—C14	117.48 (16)	C20—C21—C16	119.31 (17)
C10—C9—N1	121.61 (16)	C14—C13—C12	121.78 (16)
C14—C9—N1	120.86 (16)	C14—C13—H13A	119.1
C15—N2—C21	111.73 (15)	C12—C13—H13A	119.1
C17—C16—C21	121.22 (18)	O4—C4—Fe2	179.4 (2)
C17—C16—S3	129.47 (15)	O5—C5—Fe2	176.98 (18)
			
C4—Fe2—Fe1—C2	5.43 (9)	C3—Fe1—S2—C8	−139.75 (9)
C5—Fe2—Fe1—C2	−86.40 (9)	S1—Fe1—S2—C8	53.70 (6)
C6—Fe2—Fe1—C2	145.66 (15)	Fe2—Fe1—S2—C8	106.44 (6)
S2—Fe2—Fe1—C2	−168.97 (6)	C2—Fe1—S2—Fe2	24.94 (15)
S1—Fe2—Fe1—C2	82.74 (6)	C1—Fe1—S2—Fe2	−149.00 (6)
C4—Fe2—Fe1—C1	−118.59 (14)	C3—Fe1—S2—Fe2	113.81 (7)
C5—Fe2—Fe1—C1	149.58 (13)	S1—Fe1—S2—Fe2	−52.736 (15)
C6—Fe2—Fe1—C1	21.64 (19)	C16—S3—C15—N2	0.16 (14)
S2—Fe2—Fe1—C1	67.01 (12)	C16—S3—C15—C12	179.08 (14)
S1—Fe2—Fe1—C1	−41.28 (12)	C7—N1—C9—C10	−170.21 (16)
C4—Fe2—Fe1—C3	100.90 (10)	C8—N1—C9—C10	−11.7 (2)
C5—Fe2—Fe1—C3	9.07 (9)	C7—N1—C9—C14	12.7 (2)
C6—Fe2—Fe1—C3	−118.87 (16)	C8—N1—C9—C14	171.20 (16)
S2—Fe2—Fe1—C3	−73.50 (7)	C12—C15—N2—C21	−178.63 (15)
S1—Fe2—Fe1—C3	178.20 (7)	S3—C15—N2—C21	0.26 (19)
C4—Fe2—Fe1—S2	174.40 (7)	C15—S3—C16—C17	179.4 (2)
C5—Fe2—Fe1—S2	82.57 (6)	C15—S3—C16—C21	−0.52 (14)
C6—Fe2—Fe1—S2	−45.37 (14)	C9—N1—C7—S1	90.84 (17)
S1—Fe2—Fe1—S2	−108.29 (2)	C8—N1—C7—S1	−69.50 (18)
C4—Fe2—Fe1—S1	−77.30 (7)	Fe1—S1—C7—N1	71.07 (14)
C5—Fe2—Fe1—S1	−169.14 (6)	Fe2—S1—C7—N1	−1.10 (16)
C6—Fe2—Fe1—S1	62.93 (14)	N2—C15—C12—C13	5.1 (2)
S2—Fe2—Fe1—S1	108.29 (2)	S3—C15—C12—C13	−173.67 (13)
C2—Fe1—S1—C7	150.98 (9)	N2—C15—C12—C11	−174.37 (17)
C1—Fe1—S1—C7	51.72 (9)	S3—C15—C12—C11	6.8 (2)
C3—Fe1—S1—C7	−113.8 (3)	C13—C12—C11—C10	−0.9 (3)
S2—Fe1—S1—C7	−54.99 (6)	C15—C12—C11—C10	178.58 (16)
Fe2—Fe1—S1—C7	−107.45 (6)	C9—N1—C8—S2	−93.06 (18)
C2—Fe1—S1—Fe2	−101.57 (6)	C7—N1—C8—S2	67.16 (18)
C1—Fe1—S1—Fe2	159.17 (7)	Fe2—S2—C8—N1	5.80 (16)
C3—Fe1—S1—Fe2	−6.3 (2)	Fe1—S2—C8—N1	−66.87 (15)
S2—Fe1—S1—Fe2	52.460 (15)	C21—C16—C17—C18	−0.5 (3)
C4—Fe2—S1—C7	−151.33 (10)	S3—C16—C17—C18	179.55 (17)
C5—Fe2—S1—C7	126.56 (15)	C21—C20—C19—C18	0.7 (3)
C6—Fe2—S1—C7	−54.26 (9)	C12—C11—C10—C9	−0.2 (3)
S2—Fe2—S1—C7	48.83 (7)	C14—C9—C10—C11	1.4 (3)
Fe1—Fe2—S1—C7	101.47 (7)	N1—C9—C10—C11	−175.78 (16)
C4—Fe2—S1—Fe1	107.20 (7)	C16—C17—C18—C19	0.2 (3)
C5—Fe2—S1—Fe1	25.09 (14)	C20—C19—C18—C17	−0.4 (3)
C6—Fe2—S1—Fe1	−155.72 (6)	C10—C9—C14—C13	−1.6 (3)
S2—Fe2—S1—Fe1	−52.634 (16)	N1—C9—C14—C13	175.70 (16)
C4—Fe2—S2—C8	−117.92 (19)	C15—N2—C21—C20	179.26 (17)
C5—Fe2—S2—C8	154.81 (9)	C15—N2—C21—C16	−0.7 (2)
C6—Fe2—S2—C8	58.28 (9)	C19—C20—C21—N2	179.07 (18)
S1—Fe2—S2—C8	−50.54 (7)	C19—C20—C21—C16	−1.0 (3)
Fe1—Fe2—S2—C8	−103.10 (7)	C17—C16—C21—N2	−179.15 (18)
C4—Fe2—S2—Fe1	−14.81 (18)	S3—C16—C21—N2	0.79 (19)
C5—Fe2—S2—Fe1	−102.08 (6)	C17—C16—C21—C20	0.9 (3)
C6—Fe2—S2—Fe1	161.38 (6)	S3—C16—C21—C20	−179.16 (14)
S1—Fe2—S2—Fe1	52.558 (15)	C9—C14—C13—C12	0.4 (3)
C2—Fe1—S2—C8	131.37 (16)	C11—C12—C13—C14	0.8 (3)
C1—Fe1—S2—C8	−42.57 (9)	C15—C12—C13—C14	−178.70 (16)
